# Epitaxial Grown Carbon Nanotubes Reinforced Pyrocarbon Matrix in C/C Composites with Improved Mechanical Properties

**DOI:** 10.3390/ma14216607

**Published:** 2021-11-02

**Authors:** Ningkun Liu, Lingjun Guo, Gang Kou, Yunyu Li, Xuemin Yin

**Affiliations:** State Key Laboratory of Solidification Processing, Carbon/Carbon Composites Research Center, Northwestern Polytechnical University, Xi’an 710072, China; lnk5080223@mail.nwpu.edu.cn (N.L.); koug1255@163.com (G.K.); liyunyu@xpu.edu.cn (Y.L.)

**Keywords:** carbon nanotubes, epitaxial growth, carbon/carbon composites, Raman, mechanical properties

## Abstract

In order to achieve the highly efficient preparation of high-performance carbon/carbon (C/C) composites, epitaxial grown carbon nanotubes (CNTs) and a pyrocarbon matrix were simultaneously synthesized to fabricate CNT-reinforced C/C composites (CC/C composites). With precise control of the temperature gradient, CNTs and the pyrocarbon matrix could grow synchronously within a 2D needle-punched carbon fiber preform. Surprisingly, the CNTs remained intact within the pyrocarbon matrix at the nano-level, and the CNT-reinforced nano-pyrocarbon matrix was compact, with virtually no gaps and pores, which were tightly connected with the carbon fibers without cracks. Based on the results of Raman analysis, there is less residual stress in the CNT-reinforced pyrocarbon matrix and carbon fibers, and less of a mismatch between the coefficient and thermal expansion. Additionally, CC/C composites fabricated by this method could achieve a low density, open porosity with a large size, and improved mechanical properties. More importantly, our work provides a rational design strategy for the highly efficient preparation and structural design of high-performance CNT-einforced C/C composites.

## 1. Introduction

Carbon-fiber-reinforced pyrocarbon (C/C) composites, which have many superior characteristics (low density, high specific strength and modulus, excellent anti-friction and anti-wear, low ablation ratio, good thermal shock resistance and steady strength above 2200 °C), have been the prime structural materials within aeronautics and astronautics [1,2,3]. However, with the rapid development of new spacecraft, C/C composites with higher mechanical properties, low density, low production cost and highly efficient production are expected. As such, research on lowering the density of C/C composites while maintaining their mechanical strength has drawn much attention. This research promotes a promising train of thought which introduces carbon nanotubes (CNTs) into C/C composites with a view to improving their mechanical properties [4,5,6,7]. Ergo, the fabricated C/C composites could have a lower density because of the hollow CNTs and achieve increased mechanical properties [8,9,10]. Moreover, the increased reinforcement and structure of CNTs could accelerate the densification process of fabricating C/C composites [11,12,13].

However, the reported methods usually introduce ready-made CNTs into the carbon fiber preform first, rather than CNTs being grown in-situ on carbon fibers before the densification process [10,11,12,13,14,15,16]. The common practice was introducing already-made CNTs into carbon fiber preform before the densification process. This would create a much more complex environment in which the chemical vapor densification (CVD) process of the pyrocarbon matrix would be rendered uncontrollable. Although the introduction of CNTs could provide more active sites for the deposition of pyrocarbon to accelerate the densification process, it increases the diffusion path and resistance of carbon sources. It creates more inner pores, resulting in more un-densified pores in the C/C composites. In this case, the CNTs often hinder the fabrication process, thus yielding an unsatisfactory outcome.

Here, we propose a novel method of realizing the growth of CNTs by densification, which results in the fabrication of CNT-reinforced C/C composites (CC/C composites for convenience). A catalyst must be dispersed (for the growth of CNTs) at one side of the 2D needle-punched carbon fiber preform, rather than into the preform [10,17,18]. Next, the growth of a nano-pyrocarbon matrix occurs after the epitaxial growth of CNTs under the process of strictly controlled temperature gradient. The 2D needle-punched carbon fiber preform was densified by the CNT-reinforced nano-pyrocarbon matrix to fabricate CC/C composites. Benefiting from their unique structure, the fabricated CC/C composites had low density, low open porosity and improved mechanical properties. More importantly, this method proved more efficient and cost-effective than traditional methods [19,20,21,22,23,24], suggesting that new opportunities may be explored regarding the efficient preparation and structural design of high-performance C/C composites.

## 2. Materials and Methods

### 2.1. Preparation of C/C and CC/C Composites

**Figure 1:**
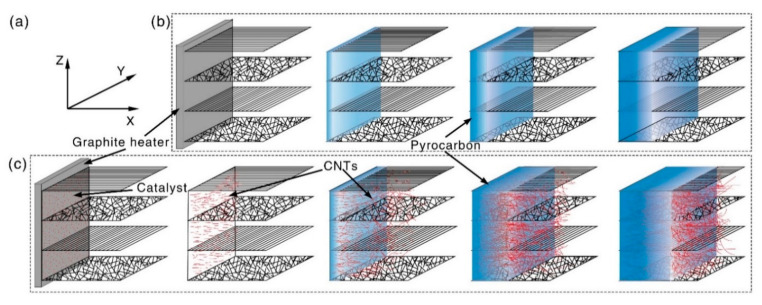
(**a**) Directions of the layered 2D needle-punched carbon fiber preform, the densification process was along X axis; Densification process of (**b**) traditional C/C composites and (**c**) CC/C composites.

A 2D needle-punched carbon fiber preform with a density of 0.4 g/cm^3^ was used initially to prepare CC/C composites and traditional C/C composites. The carbon fiber preform was fabricated by over-lapping layers of 0° non-woven carbon fiber cloth, short-cut fiber web and 90° non-woven carbon fiber cloth. These layers were repeated and vertically needle-punched (Z axis), as shown in Figure 1. The catalyst was located at one side of the 2D needle-punched carbon fiber preform adjacent to the graphite heater, as shown in Figure 1c. A controlled temperature gradient was manipulated by the electric current passed through the heater of a thermal gradient chemical vapor deposition machine. Due to the thermal gradient, the temperature decreased along the X axis; therefore, CNTs formed and grew up when the temperature was low and was not high enough to generate pyrocarbon matrix. With the temperature of the heater increasing, the low-temperature zone moved forward along the X axis, and the increased temperature promoted the generation of the pyrocarbon matrix required to densify the 2D needle-punched carbon fiber preform containing the previously grown CNTs. By ensuring the temperature gradually moved along the X axis, the whole 2D needle-punched carbon fiber preform could be densified into fabricated CC/C composites. The densification temperature was 705–1155 °C. Natural gas (with methane content 99%) was entered as the precursor of carbon, and its volume flow rate was 3–15 L/min. The densification process proceeded along the X axis. For comparison, pure C/C composites without CNTs were also prepared utilizing the same process, and the schematic diagram of the densification process is shown in Figure 1b.

### 2.2. Mechanical Properties

Mechanical properties of the fabricated CC/C composites and C/C composites were determined by a three-point bending test, compression test, and shear test on a SANS universal mechanical machine (CMT5304-30 kN and CMT5304-1 kN) at a constant speed of 0.2 mm/min. Rectangular bars with a dimension of 30 mm × 5 mm × 2 mm were cut from the composites to carry out the three-point bending test, and the span was 20 mm. Meanwhile, cubes of 10 mm × 6 mm × 6 mm and 5 mm × 4 mm × 4 mm were used to implement the compression test and shear test, respectively. The cutting and loading directions were defined as follows: the direction perpendicular to the over-lapping layers of 2D needle-punched carbon felts was labelled Z direction, and the direction parallel to the over-lapping layers of 2D needle-punched carbon felts was labelled X or Y direction.

### 2.3. Materials Characterizations

The density of the fabricated samples was measured by the Archimedes method in water. A Leica DMLP polarized light microscope (PLM, DMLP) was used to observe the textures of the obtained composites. The fracture surface morphology and microstructure were characterized by scanning with electron microscopes (SEM, Tescan Mira 3, Czech and ZEISS Supra 55) and transmission electron microscopes (TEM, FEI Tecnai F30 G2). Micro residual stress in the composites was analyzed by Raman G peak mapping using Raman spectroscopes (InVia, Renishaw, He-Ne laser, 532 nm).

## 3. Results

**Figure 2:**
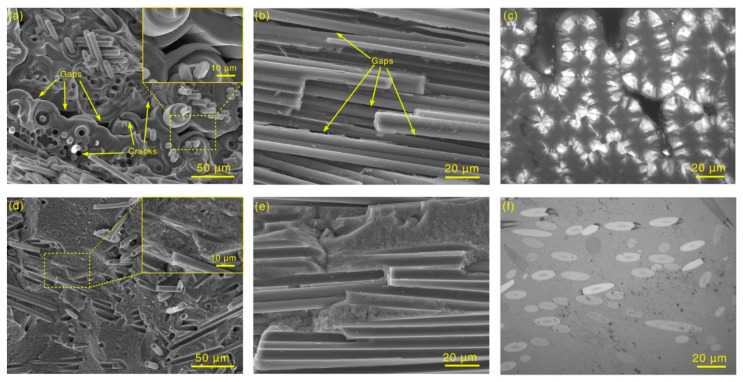
SEM images and PLM images of (**a**–**c**) C/C and (**d**–**f**) CC/C composites: (**a**,**d**) SEM images of carbon fibers perpendicular to fracture surface; (**b**,**e**) SEM images of carbon fibers parallel to fracture surface; (**c**,**f**) PLM images.

In the fracture morphologies (Figure 2a,b) and polarized light microscope (PLM) image (Figure 2c) of the traditional C/C composites, although the density reached 1.76 g/cm^3^ (with an open porosity of 6.5%), there were still many gaps that were not fully densified during the fabrication process, which were distributed between carbon fibers. The pyrocarbon matrix had a layered structure (the insert in Figure 2a), and cracks could be found in the annular layers. In Figure 1b, the gap lengths increased to hundreds of microns, representing potential cracks during fracturing. The PLM image (Figure 2c) illustrates that the pyrocarbon matrix in traditional C/C composites showed intense optical activity (high and medium texture), which was different from that of the carbon fibers [20,21,25,26], and indicates very different matrix structures between the fibers. The various structures of carbon usually have different properties. For example, the coefficient of thermal expansion (CTE) of high- and medium-texture pyrocarbon is larger than 10 × 10^−6^/°C [27], but it is less than 2 × 10^−6^/°C for carbon fibers [28]. As a result, the mismatching of CTE, induced by the different structures, could cause numerous cracks to emerge throughout the layered pyrocarbon matrix and between carbon fibers and pyrocarbon matrix [26,29,30], which will significantly influence the properties of C/C composites.

Although the density and open porosity of CC/C composites were less than 1.75 g/cm^3^ and 3.4%, respectively, there were few pores within them. Carbon fibers were compacted into the matrix, and no cracks or gaps were found in the CNT-reinforced pyrocarbon matrix (Figure 2d–f). No layered structure was found in the CC/C composites, and the matrix showed no growth orientation. In the fiber bundle (Figure 2e), the CNT-reinforced pyrocarbon matrix can completely fill the gaps between carbon fibers, and no cracks or gaps form between the fibers and the matrix. In the magnified fracture morphology (insert in Figure 2d), many micro-nano holes, which can be attributed to the CNT pull-out and the unfilled gaps between CNTs and the carbon matrix, could be found in the CNT-reinforced matrix. They were uniformly distributed throughout the matrix. The PLM image (Figure 2f) shows that the CC/C composites are compact, and both the CNT-reinforced pyrocarbon matrix and carbon fibers displayed low optical activity (low texture and isotropic). Therefore this indicates that they have a similar microstructure, which can reduce the mismatch of CTE between carbon fibers and the matrix.

**Figure 3:**
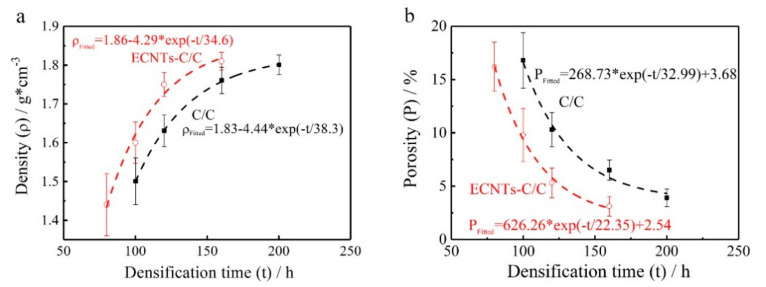
(**a**) Density and fitted limit density of CC/C and traditional C/C composites; (**b**) Porosity and fitted limit porosity of CC/C and traditional C/C composites.

Benefitting from the synchronous growth of CNTs and a pyrocarbon matrix, the CC/C composites can be fabricated more efficiently than C/C composites, and traditional CNT-reinforced C/C composites. As shown in Figure 3, the density of CC/C composites is much higher than that of C/C composites with the same densification time, and for the same density, the densification time of CC/C composites is shorter than that of C/C composites. In addition, for the traditional CNT-reinforced C/C composites, though the densification time is equal to that of the C/C composites, it is more costly timewise to carry out the growth of CNTs before the densification process. As a result of the impact of CNTs, its densification processing is more difficult than C/C composites, and more pores would be left in the fabricated CNT-reinforced C/C composites. However, the method proposed by this research can minimize the adverse effect of CNTs on the densification process and achieve an increased densification rate. As such, the proposed method is more efficient and low-cost than the traditional densification method. With a prolonged densification time, the fitted limit density of CC/C composites (1.86 g/cm^3^) is also larger than that of C/C composites (1.83 g/cm^3^).

**Figure 4:**
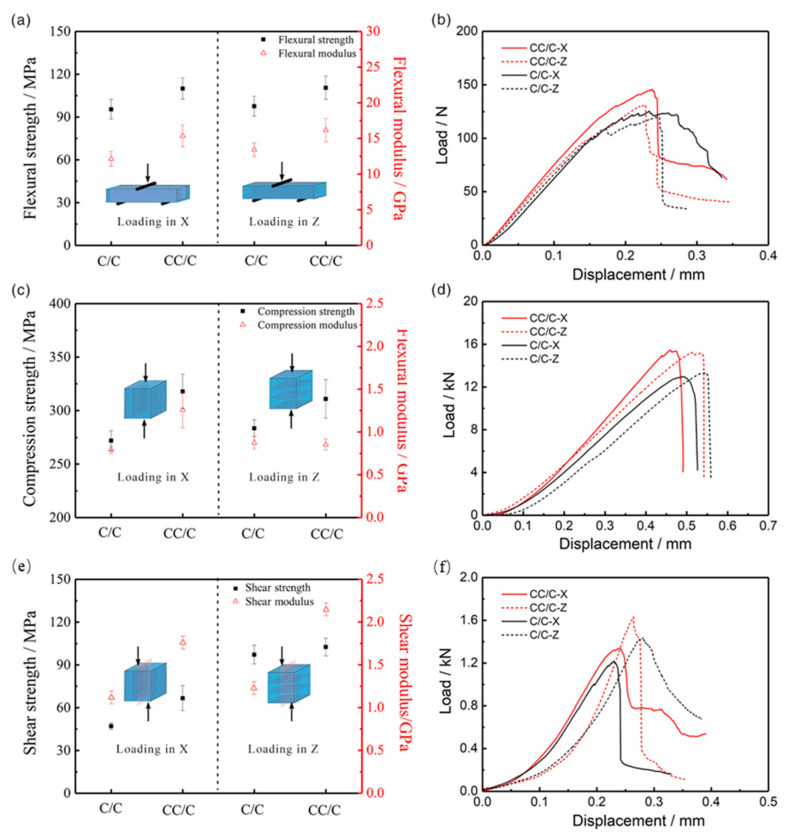
Mechanical properties of CC/C and traditional C/C composites: (**a**,**b**) Three-point bending test; (**c**,**d**) Compression test; (**e**,**f**) Shear test.

In general, C/C composites with a higher density usually exhibit higher mechanical strength [30]. As shown in Figure 4, in this work, CC/C composites with a lower density of 1.75 g/cm^3^ exhibited a higher mechanical strength than those of C/C composites with a higher density of 1.80 g/cm^3^. The flexural strength (at X, Z directions), compression strength (at X, Z directions) and shear strength (at X, Z directions) were increased by 15.2%, 13.2%, 16.9%, 9.7%, 41.9%, 5.5%, respectively. This indicates that the CNT-reinforced pyrocarbon matrix, induced by a synchronous growth method, greatly improves the mechanical strength of CC/C composites. The hollow CNTs simultaneously decrease the density. Corresponding to the strength, the modulus of CC/C composites is also higher than that of C/C composites. This illustrates that the CNTs reinforced pyrocarbon matrix has a higher strength and modulus than the pyrocarbon matrix in C/C composites. Moreover, the load-displacement curves show little difference between the two composites, showing similar fracture processing.

**Figure 5:**
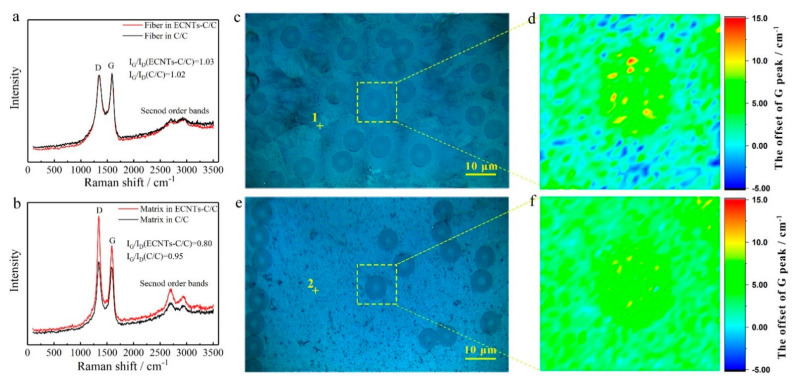
Raman spectra analysis: (**a**) Carbon fibers in C/C and CC/C composites before densification; (**b**) Matrix in C/C (Point 1) and CC/C (Point 2) composites after densification process; (**c**,**e**) Optical micrographs of C/C and CC/C composites; (**d**,**f**) Raman mapping of the shift of G peak, reflecting the stress distribution in carbon fiber in C/C and CC/C composites.

Since the fabrication temperature was higher than 1000 °C and the CTE of pyrocarbon is much larger than that of carbon fibers along the radial direction, the intrinsic stress caused by the mismatching of CTE was inevitable between carbon fibers and the pyrocarbon matrix. Usually, Raman spectroscopy is an effective method to analyze the structure of carbon-based materials [31,32], and Raman mapping of the shift in the G peak is used to evaluate the stress distribution in and around carbon fibers [33]. As displayed in Figure 5a, before densification, there was no obvious difference in the Raman spectra of carbon fibers, and no stress was applied to the carbon fiber. After densification, the carbon fibers and matrix suffered the intrinsic stress caused by the mismatching of CTEs of the pyrocarbon matrix and carbon fibers. The G peak could shift toward higher (compressive stress) or lower frequencies (tensile stress) [34,35,36]. Figure 5b shows that the intensity ratio of D and G peaks (I_D_/I_G_) of CC/C composites is much larger than that of C/C composites, indicating that carbon atoms in the matrix of CC/C composites are much closer to a short-range order structure. Additionally, a 2D band of around 2700 cm^−1^ and D + G band of around 2950 cm^−1^ were detected. I_2D_/I_G+D_ are usually indicators of texture. A higher I_2D_/I_G+D_ mean a more ordered in-plane structure. Figure 5b shows that the structure of the carbon matrix of C/CC composites was more ordered than the traditional C/C composites. This indicates that the CNTs in the matrix improve the texture of C/CC composites. The results of the second order bands fit well with the first order bands. However, compared to that of the C/C composites, they have shifted a little and, therefore, could not be used as the reference point to evaluate the intrinsic stress. Therefore, the G peak of carbon fibers before densification (1590.4 cm^−1^ in Figure 5a) was used as the reference point to evaluate the intrinsic stress within the fabricated composites. Figure 5c,d demonstrates that the carbon fiber in C/C composites shows a significant difference from the matrix and the G peak has increased, indicating that the carbon fiber has suffered strong compressive stress. However, for the CC/C composites (Figure 5e,f), little difference is found between the carbon fiber and the matrix, indicating a smaller shift in the G peak for carbon fiber and low intrinsic stress in the fabricated CC/C composites.

**Figure 6:**
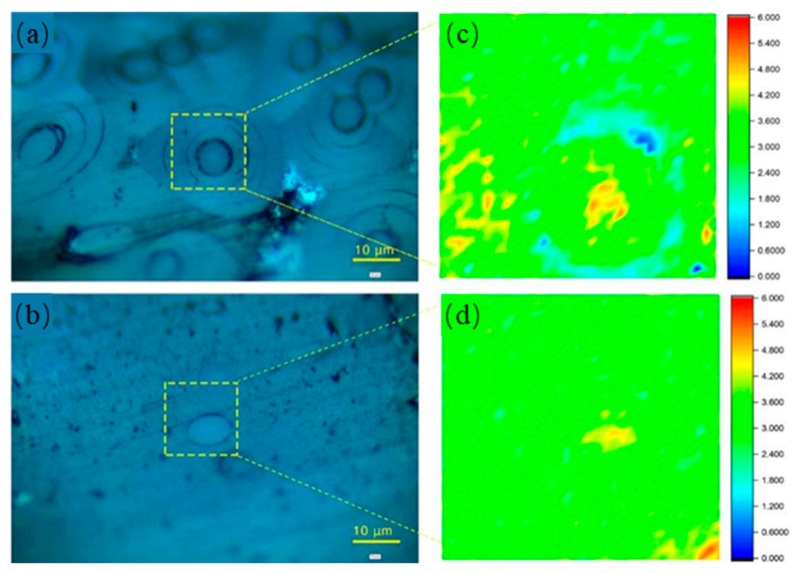
Raman spectra analysis after thermal shock test of (**a**,**c**) C/C and (**b**,**d**) CC/C composites: (**a**,**b**) Optical micrographs; Raman mapping of the shift in G peak, reflecting the stress distribution in carbon fiber.

**Figure 7:**
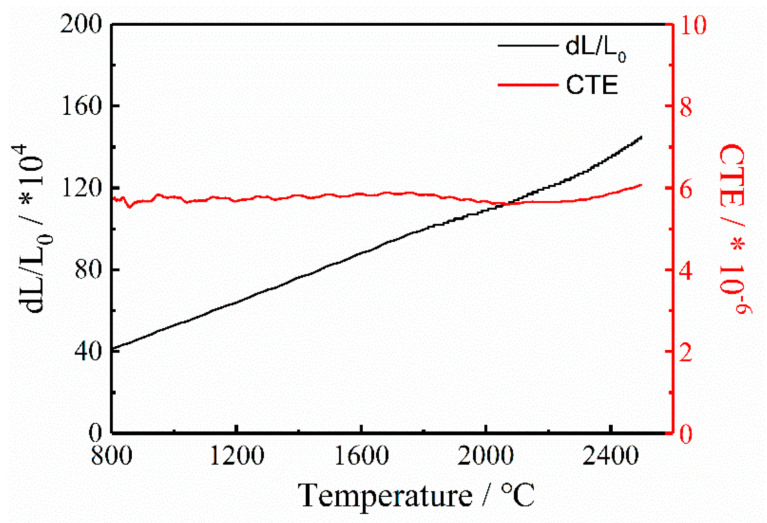
CTE of the CNTs reinforced pyrocarbon matrix of CC/C composites.

In the thermal shock test, because of the different structures in C/C composites, cracks induced by the mismatching of CTE (carbon fibers: <2 × 10^−6^/°C [28] and pyrocarbon matrix: >10 × 10^−6^/°C [27]) would form in the composites. As shown in Figure 6a, cracks form in the pyrocarbon matrix after the thermal shock test, and carbon fibers are separated from the matrix. In Figure 6b, there is little difference in the shift in the G peak between the carbon fiber and the pyrocarbon matrix. This indicates that the intrinsic stress in carbon fibers is released during the thermal shock test due to of the formation of cracks. However, in CC/C composites (Figure 6c), no cracks form in the CNT-reinforced pyrocarbon matrix, and no obvious gaps form around the carbon fibers. The CTE of the CNT-reinforced pyrocarbon matrix is 6 × 10^−6^/°C (Figure 7), which is also higher than that of carbon fibers, but the mismatching of CTE between the two different structures is decreased. The Raman mapping of the shift in the G peak also shows ittle difference between the CNT-reinforced pyrocarbon matrix and carbon fibers in the CC/C composites (Figure 6d). As a result, less intrinsic stress exists in the CC/C composites, and fewer cracks are formed after the thermal shock test.

**Figure 8:**
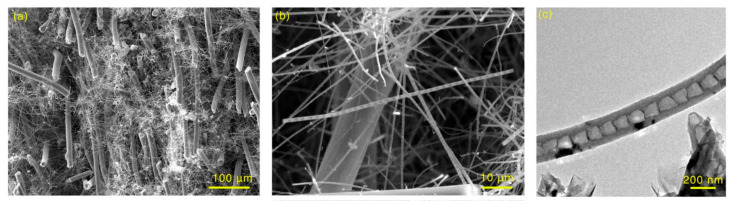
(**a**,**b**) SEM and (**c**) TEM images of CNTs in 2D needle-punched carbon fiber preform.

During the fabrication process of the CC/C composites, the temperature gradient, is not hot enough for pyrocarbon to be generated. Furthermore, due to the catalytic growth of the catalysts, CNTs can initiate and grow prematurely between carbon fibers within a limited area (Figure 8a,b). They randomly distribute within the carbon preform. The CNTs show a bamboo-like microstructure [37,38,39,40], and the typical outer and inner diameter for CNTs is ~200 and 100 nm (Figure 8c). Due to the in-site growth of CNTs, more sites are formed for the deposition of pyrocarbon, which could accelerate the densification rate of C/CC composites. Thus, the densification rate of C/CC composites would be much higher than C/C with the same process. As a result, the density of the fabricated CC/C composites was much higher than that of the traditional C/C composites with the same densification time (Figure 3).

**Figure 9:**
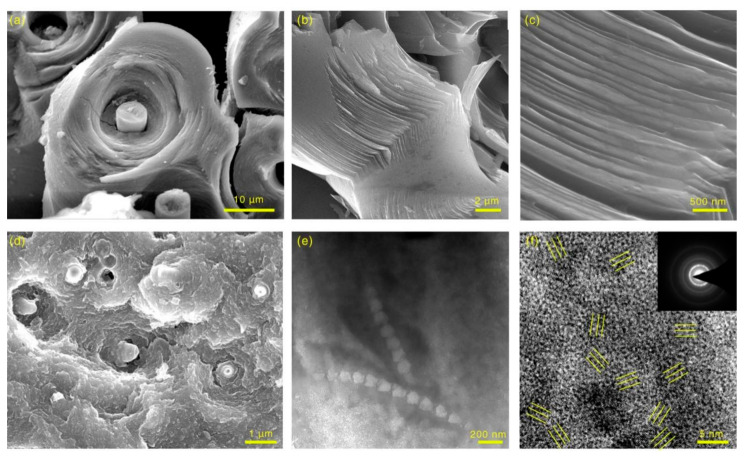
(**a**–**c**) Fracture morphology of C/C composites; (**d**) Fracture morphology of CC/C composites; (**e**) TEM image of the CNTs reinforced pyrocarbon matrix in CC/C composites; (**f**) HRTEM image of Pyrocarbon matrix in the CC/C composites (Insert f is the SAED).

The SEM images (Figure 9a–c) of the fracture show that the pyrocarbon matrix in C/C composites forms layered structures around the carbon fibers, and many gaps separate them. The reason for this is that, perpendicular to the direction of the layered pyrocarbon, the CTE is much higher than that of the parallel direction and carbon fibers. As a result, when it suffers thermal shock, cracks are easily generated in the layered pyrocarbon matrix. For CC/C composites (Figure 9d), many CNTs are pulled out and holes are distributed on the fracture surface. In addition, the pyrocarbon matrix completely fills the space between carbon fibers. The fracture surface of the CNT-reinforced pyrocarbon matrix is much rougher than that of C/C composites. As illustrated in the TEM image (Figure 9e), CNTs display a bamboo-like microstructure and remain intact in the matrix, which results in the mechanical properties of CC/C composites. As shown in the high-resolution TEM (HRTEM) image and the selected area electron diffraction (SAED) pattern (Figure 9f), the pyrocarbon matrix is composed of randomly oriented micro- and nano-crystals and amorphous carbon. In this way, the hollow CNTs in the pyrocarbon matrix can increase the mechanical properties and reduce the density of the C/C composites.

## 4. Conclusions

In conclusion, we report a novel method of fabricating CNT-reinforced C/C composites by densifying a 2D needle-punched carbon fiber preform with the synchronous growth of CNTs and a pyrocarbon matrix. During the densifying process, CNTs form at the relatively low-temperature zone ahead of the densifying zone, so they have little influence on the gas transmission of carbon sources in a 2D needle-punched carbon fiber preform. The preform ensures that the carbon sources can diffuse into the densifying zone that includes CNTs and carbon fibers, avoiding the densification that occurs in a wide space, which could create many holes in C/C composites. Compared with the fabrication process of traditional C/C and CNT-reinforced C/C composites, the CC/C composites fabricated by the proposed method have low density, low open porosity, and improved mechanical properties, which makes the fabrication of high-performance CNT-reinforced C/C composites more efficient. This research provides an impressive and novel structural design strategy for the highly efficient preparation of high-performance C/C composites, which could potentially be used to manufacture aircraft materials that need to withstand high temperatures.

## Data Availability

Data Sharing is not applicable.
